# A Pilot Randomized Clinical Trial: Oral Miltefosine and Pentavalent Antimonials Associated With Pentoxifylline for the Treatment of American Tegumentary Leishmaniasis

**DOI:** 10.3389/fcimb.2021.700323

**Published:** 2021-07-01

**Authors:** Sofia Sales Martins, Daniel Holanda Barroso, Bruna Côrtes Rodrigues, Jorgeth de Oliveira Carneiro da Motta, Gustavo Subtil Magalhães Freire, Ledice Inácia de Araújo Pereira, Patrícia Shu Kurisky, Ciro Martins Gomes, Raimunda Nonata Ribeiro Sampaio

**Affiliations:** ^1^ Pós-Graduação de Ciências da Saúde da Faculdade de Ciências Saúde, Universidade de Brasília, Brasília, Brazil; ^2^ Hospital Universitário de Brasília, Universidade de Brasília, Brasília, Brazil; ^3^ Pós-Graduação de Ciências Médicas da Faculdade de Medicina, Universidade de Brasília, Brasília, Brazil; ^4^ Laboratório de Dermatomicologia da Faculdade de Medicina, Universidade de Brasília, Brasília, Brazil; ^5^ Departamento de Doenças Infecciosas, Hospital de Doenças Tropicais Dr. Anuar Auad (HDT), Goiânia, Brazil

**Keywords:** pentavalent antimonial, randomized clinical trial, pentoxifylline, miltefosine, cutaneous leishmaniasis, mucosal leishmaniasis, American tegumentary leishmaniasis

## Abstract

**Introduction:**

American tegumentary leishmaniasis (ATL), which can present as either cutaneous (CL) or mucosal leishmaniasis (ML), is endemic in South America, and first-line antimonial treatments are known for their wide range of adverse effects (AEs). Growing reports of drug resistance increase the urgency of the need for better treatment options. The objective of this pilot clinical trial was to assess the efficacy of and AEs associated with the oral combination of miltefosine and pentoxifylline based on a *post hoc* analysis.

**Methods:**

A pilot, randomized, open-label clinical trial was performed. The experimental group (M+P) received 50 mg twice a day (BID) miltefosine and 400 mg three times a day (TID) pentoxifylline, and the control group (A+P) received 20 mg Sb+V/kg/day intravenously and 400 mg TID pentoxifylline. Patients with ML received treatment for 28 days, and patients with CL received treatment for 20 days.

**Results:**

Forty-three patients were included: 25 with ML and 18 with CL caused by *L.(V.) braziliensis*. AEs were more frequent in the A+P group (p=0.322), and there was a need for treatment interruption due to severe AEs (p=0.027). Patients with CL had a higher chance of achieving a cure (p=0.042) and a higher risk of AEs (p=0.033). There was no difference in the chance of a cure based on the treatment (p=0.058).

**Conclusion:**

In this pilot randomized clinical trial, M+P treatment and A+P treatment yielded similar cure rates, and the former was associated with a lower risk of AEs. Future studies with more patients and longer follow-up are recommended.

## Introduction

Between 2001 and 2017, there were 940.396 new cases of tegumentary leishmaniasis, including both the cutaneous (CL) and mucous (ML) forms, in the Americas, with an annual mean of 55.317 cases. These cases were reported by 17 of the 18 endemic countries on the continent, and 72.6% of the cases were in Brazil. The incidence of ML was 3.78% of all LTA cases in Brazil (Organização Pan-Americana da Saúde, 2019).

Active drug treatment is the main form of disease control, although it does not affect asymptomatic infected individuals. Many drugs have been used to treat leishmaniasis, but first-line therapy with pentavalent antimonials (PAs) has not changed for decades. PAs have been used in the Americas since the 1940s. PAs are known for their wide range of adverse effects (AEs), leading to treatment interruptions, hepatic and cardiac alterations and even death, and the rate of drug resistance is increasing. Other second-line therapies, such as amphotericin and pentamidine, are also injectable and are associated with significant AEs. Currently, cure rates vary from 70 to 90% in patients with CL and from 30% to 91% in those with ML (Chakravarty and Sundar, 2019).

A 40% rate of treatment failure has been reported in patients treated with intravenous PAs alone for infection with *Leishmania (V) braziliensis* (Ventin et al., 2018). Therapeutic failure is becoming increasingly common in Brazil, especially in patients with *L. (V) braziliensis* infections (Ponte-Sucre et al., 2017; Rugani et al., 2018). The underlying mechanisms are not yet clear but seem to be related to parasitological drug resistance and the lack of a host immune response. The current treatment recommended by the Brazilian Health Ministry for LC is 10 to 20 mg SbV/kg/day for 20 days and that for LM is 20 mg SbV/kg/day combined with 400 mg pentoxifylline three times per day for 30 days (Ministério da Saúde, 2017).

In this context, treatments involving a combination of oral drugs are interesting alternatives with the aim of increasing efficacy, reducing AEs and increasing treatment adherence in patients with leishmaniasis (Chakravarty and Sundar, 2019). Combining oral drugs is already a successful treatment strategy for other infectious diseases caused by intracellular microorganisms, such as tuberculosis and leprosy, and is known to reduce drug resistance.

Miltefosine is the first oral drug with efficacy against leishmaniasis, and it has been used since 2002 for the treatment of both visceral (Sundar et al., 2002) and mucocutaneous leishmaniasis. It affects the phospholipid membrane integrity and mitochondrial function of microorganisms (Sundar et al., 2002; Soto et al., 2004; Chrusciak-Talhari et al., 2011; Sampaio et al., 2019). It also has an indirect effect by acting as an immunomodulator against Leishmania, promoting the production of IFN-γ, TNF-α and IL-12 and stimulating phagocytosis and the Th1 pathway (Santarem et al., 2014).

Miltefosine is usually well tolerated, with mild gastric and hepatic AEs; however, it is known to be a teratogenic drug (Dorlo et al., 2012). Unfortunately, there are already reports of resistance to miltefosine when it is used alone for both visceral and tegumentary leishmaniasis. *In vitro*, *L. (V.) braziliensis* had a 68% rate of resistance to monotherapy with miltefosine (Fernández et al., 2014), and one treatment course was sufficient for the development of resistance (Berman, 2008).

Pentoxifylline is a methylxanthine with anti-inflammatory effects that suppresses TNF-α gene transcription, increases nitric oxide production and decreases leukocyte migration and adhesion. It is known to have an adjuvant immunologic effect when associated with pentavalent antimony for the treatment of mucocutaneous leishmaniasis (González et al., 2009; Santarem et al., 2014; Burza et al., 2018). It also has a nephroprotective effect when associated with PAs (Santarem et al., 2014). In C5BL/6 mice infected with *L. (L.) amazonensis*, pentoxifylline combined with antimonials was able to reduce macrophage vacuolization and induce more effective parasite destruction (Santarem et al., 2014).

Considering the urgent need for clinical trials of treatments for ATL (Ponte-Sucre et al., 2017; Pinart et al., 2020), especially neglected ML, the objective of this pilot clinical trial was to assess the efficacy and toxicity of the oral combination of miltefosine and pentoxifylline and the standard treatment, consisting of intravenous PAs and oral pentoxifylline, in an endemic region for *Leishmania (V.) braziliensis*, based on a *post hoc* analysis.

## Material and Methods

### Population and Case Definition

A pilot, open-label randomized clinical trial (RCT) was performed from August 2015 to August 2020 in two referral centers for leishmaniasis in the central region of Brazil located in the cities Brasília and Goiânia (Universidade de Brasília and Hospital Estadual de Doenças Tropicais Dr. Anuar Auad). The ATL case definition relied on the presence of cutaneous or mucosal symptoms observed on clinical examination and rhinoscopy (cutaneous ulcer, infiltrated cutaneous plaque or nodulus, progressive nasal congestion, rhinorrhea, epistaxis and destructive lesions of the nasal septum, lips and palate) associated with laboratory and epidemiological confirmation as described elsewhere (Gomes et al., 2014). TaqMan-based real-time PCR with specific *L (V.) braziliensis* probes was performed as described elsewhere (Gomes et al., 2017; Bergmann et al., 2019) as a method of diagnosis and species identification.

All eligible ATL patients were consecutively included and underwent video nasoendoscopy and cutaneous, nasal or laryngeal biopsy with histopathological evaluation. We excluded patients under 18 years of age and over 80 years of age, patients with more than 3 cutaneous lesions, patients who received any antileishmanial drugs 6 months prior to the diagnosis, and patients with severe hepatic, renal or cardiac disease, malignant neoplasia, HIV infection or Chagas disease. Due to the potential teratogenic effects of pentavalent antimonials and miltefosine, pregnant or breastfeeding women, women who were not using effective contraceptive methods were also excluded.

### Randomization

Patients were automatically randomized in blocks of 4, 6 and 8 using the online randomization system Sealed Envelop™ (Sealed Envelope Ltd. 2011) at a ratio of 1:1 into two groups (M+P or A+P).

### Interventions

The experimental group (M+P) received 50 mg twice a day (BID) miltefosine and 400 mg three times a day (TID) pentoxifylline for 28 days if they had confirmed mucosal lesions or for 20 days if they had no evidence of mucosal lesions and only had CL. Patients in the control group (A+P) received 20 mg Sb+V/kg/day up to 1215 mg/day intravenously and 400 mg TID pentoxifylline for 30 days if they had ML or for 20 days if they had CL, according to the treatment recommended by the Brazilian Ministry of Health.

### Outcomes

The primary outcome was defined as the cure of leishmaniasis. The occurrence of AEs was considered a secondary outcome. Patients were considered cured if they had complete healing (reepithelization without infiltrations or erythema) of the lesions up to 90 days after the beginning of the treatment. An additional evaluation of the curative effect was performed 180 days after the beginning of the treatment.

### Patient Follow-Up

Patients were monitored weekly to identify AEs, which were characterized as clinical, laboratory and electrocardiographic changes that occurred during treatment and had no possible causal relationship with external factors. AEs were classified as mild or severe.

The mild AEs were myalgia, arthralgia, headache, local inflammation, nausea, vomiting, dizziness, asthenia and stomachache. If they developed mild AEs, patients were monitored closely. Severe AEs were hepatic alterations with elevated transaminase levels [>2.5x upper limit of normal (ULN)], renal alterations (creatinine > 1.5x ULN), elevated levels of amylase (>1.5x ULN), anemia (hemoglobin <9.5 g/dL) and cardiac alterations with QTc interval enlargement (QTc>450 ms). In those cases, patients discontinued treatment, which was only reintroduced when the alterations were normalized.

If patients could not complete the proposed drug therapy 75 days after it was initiated due to severe or persistent AEs, they were treated with liposomal amphotericin B (LAB). Patients were followed-up at 30, 60, 90, and 180 days after the beginning of treatment and once a year thereafter.

### Statistical Analysis

Bivariate and multivariate Cox regression were used to determine the significant predictors of achieving a cure and the occurrence of AEs, and the model and the associated 95% confidence interval were constructed. Hazzard ratios (HRs) and their respective 95% confidence intervals were calculated. Multicollinearity was evaluated between the independent variables. The cutoff value of the tolerance indicator for the detection of multicollinearity was 0.603. P < 0.05 was considered significant. The analyses were conducted using SAS® 9.4 software (SAS Institute Inc., Cary, North Carolina, USA).

This RCT was registered at clinicaltrials.gov under the number CT02530697 and in the Brazilian clinical trials registry under the number RBR-72dv9n. The Brazilian ethics committee approved it in May 2015 under the number CAAE: 40068714.1.1001.5558.

## Results

**Figure 1:**
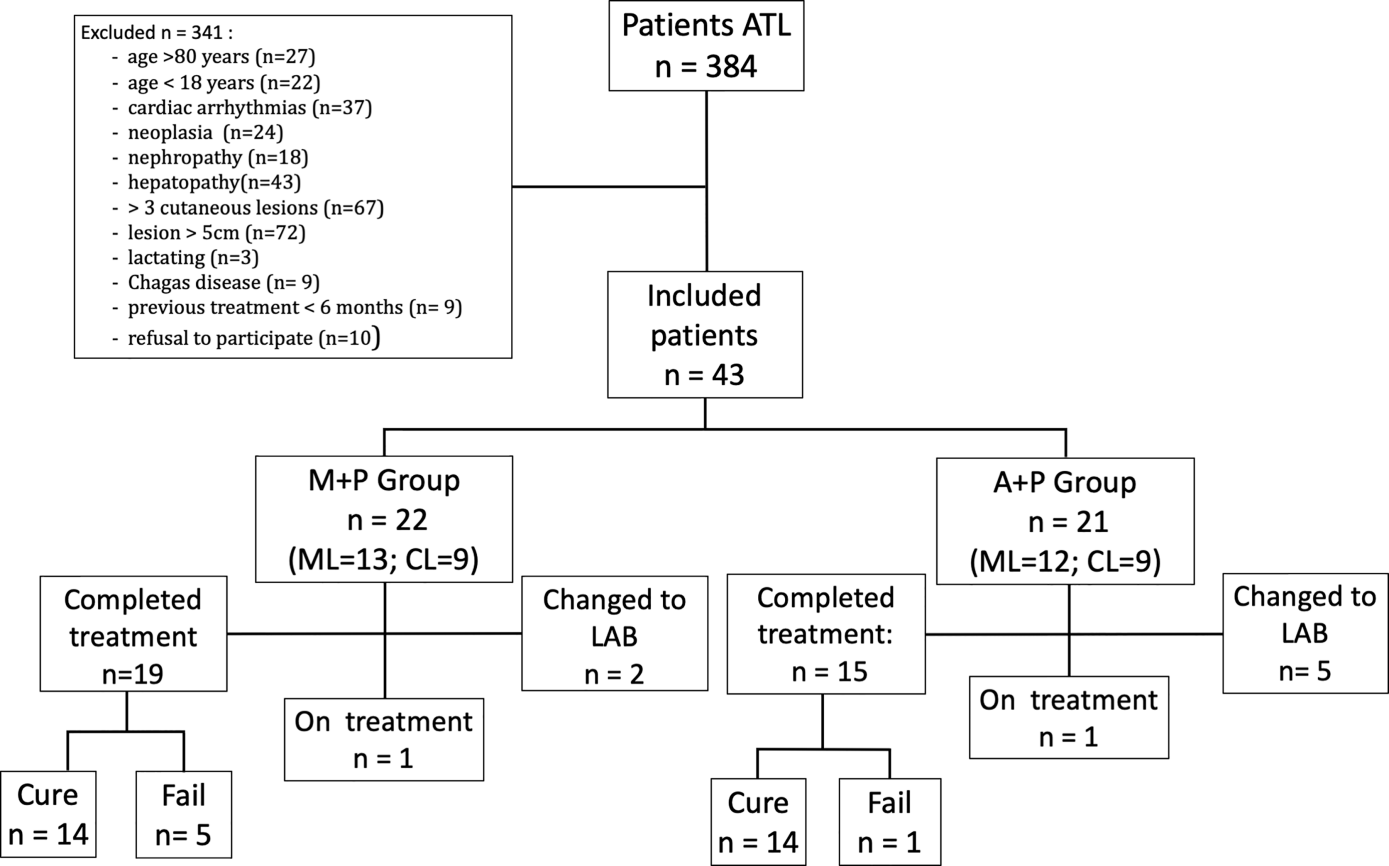
Flow diagram showing eligible patients, randomized patients and cure outcomes.

Of the 384 patients with suspected diagnoses of ATL in the pilot RCT period, 43 patients were included and randomized. Twenty-two were assigned to the M+P group and 21 to the A+P group (**Figure 1**). There were 348 patients with a confirmed diagnosis of LC, of whom 18 were included, and there were 43 patients with LM, of whom 25 were included.

### Demographic Characteristics

The mean age of all included patients was 51.8 years (ranging from 19 to 79 years), and approximately 67% of the patients were male. The time between the beginning of symptoms and the diagnosis ranged from 1 to 180 months, with a mean of 28 months. Twenty-five patients had ML and 18 had CL. Three patients had received specific treatment for leishmaniasis more than 6 months before being included in this RCT (6.9%). The number of lesions ranged from 1 to 2, but the majority of the patients had only one lesion, with a mean of 1.18. The patients’ weight ranged from 48.5 to 88 kg, with a mean of 68.7 kg. Forty-eight percent of all patients had one or more comorbidities, and the most frequent comorbidities were hypertension and diabetes mellitus. When we analyzed only the 25 patients with ML, the mean age was 58 years, and the mean time from symptom onset to diagnosis was 41 months.

There were no significant differences in the clinical and demographic characteristics (sex, weight, number of lesions, time from lesion detection to diagnosis, disease form (LC or LM), previous treatment and comorbidities) between the two groups.

### Treatment Duration and Doses

In the intention-to-treat analysis, the treatment duration varied from zero to 54 days (**Table 1**). One of the patients in the M+P group could not start treatment because after randomization, his pharyngeal lesion worsened, and he was not able to swallow the drugs. This patient was then treated with LAB.

**Table 1:** Univariate analysis of treatment outcomes.

	Group		p-value
	M+P (n = 22)	A+P (n = 21)	
	Mean (SD)	Mean (SD)	
**Treatment duration (days)**	22.5 (7.35)	26.75(14.89)	0.241
**Interruption duration (days)**	0.15 (0.67)	10.86 (16.22)	0.258
**Total treatment duration (days)**	22.63 (7.33)	37.68 (26.42)	0.014
**Dosage of Sb5+ (mg/kg/day)**	–	17.19 (2.16)	-
**Dosage of miltefosine (mg/kg/day)**	1.36 (0.35)	–	-
	**n (%)**	**n (%)**	
**Treatment interruption**	3 (13.63)	12 (57.14)	0.027
**Treatment changed to LAB**	2 (9.09)	5 (23.08)	0.229
**Complete healing 30 days from the beginning of treatment**	6 (27.27)	8 (38.09)	0.452
**Cure**	14 (69.23)	14 (66.66)	0.196
**Adverse effects**	11 (50)	19 (90.47)	0.003

Legend M+P, treatment with miltefosine and pentoxifylline; A+ P, treatment with pentavalent antimonial and pentoxifylline; SD, standard deviation; n, number of patients; Sb5+, pentavalent antimonial; LAB, liposomal; B, amphotericin.

We compared treatment duration, dosing and outcomes in the M+P and A+P groups.

Patients in the M+P group had to discontinue treatment for a mean of 0.15 days, as most patients did not interrupt treatment, while in the A+P group, the mean number of days of treatment interruption was 10.86. When we considered the entire duration of the treatment, including interruptions, the mean duration in the M+P group was 22.63 days and that in the A+P group was 37.68 days (p=0.014) (**Table 1**). The proportions of patients who needed to discontinue treatment due to AEs were 13.63% in the M+P group and 57.14% in the A+P group (p = 0.027) (**Table 1**).

The PA dosage varied from 13.96 to 19.91 mg/kg/day, with a mean of 17.19 mg/kg/day. In the M+P group, the daily miltefosine dosage varied from 1.13 mg to 1.79 mg/kg/day, with a mean of 1.36 mg/kg/day (**Table 1**).

### Curative Effect

In the univariate analysis of the curative effect 90 days after the beginning of the treatment, there was no difference between the two groups (p = 0.196) (**Table 1**). In the M+P group, 69.23% of the patients were cured; in the A+P group, 66.66% of the patients were cured. In the secondary analysis of the result 180 days after the beginning of treatment, the finding remained the same. Complete healing within 30 days from the beginning of treatment was achieved by 27% of the patients in the M+P group and 38% of the patients in the A+P group (p = 0.452) (**Table 1**).

In multivariate analysis of the curative effect, there were no significant differences between the two groups (HR = 2.44 (CI: 0.97 - 6.14); p = 0.058) based on the adjusted Cox regression model. Patients who had previously undergone antileishmanial treatment more than 6 months before had a higher cure rate (HR = 7.91 (CI: 1.27 – 49.41); p = 0.026), as did patients with only cutaneous lesions (HR = 7.73 (CI: 1.07 – 55.72); p = 0.042). (**Table 2**).

**Table 2:** Multivariate analysis results showing hazard ratios for cure and adverse effects in patients treated with miltefosine and pentoxifylline and in patients treated with pentavalent antimonial and pentoxifylline.

Cure	Crude Hazard Ratio		Adjusted Hazard Ratio	
	PR (CI 95%)	p-value	RR (CI 95%)	p-value
**Specific previous treatment**		0.5158		0.0269
No	1	–	1	–
Yes	1.63 (0.37; 7.08)	0.5158	7.91 (1.27; 49.41)	0.0269
**Clinical presentation**		0.0161		0.0425
Mucosal	1	–	1	–
Cutaneous	3.27 (1.25; 8.58)	0.0161	7.73 (1.07; 55.72)	0.0425
**Treatment Group**		0.0949		0.0589
M + P	1	–	1	–
A + P	1.99 (0.89; 4.44)	0.0949	2.44 (0.97; 6.14)	0.0589
**Adverse effects**	**Crude Hazard Ratio**		**Adjusted Hazard Ratio**	
**Specific previous treatment**		0.4608		0.0090
No	1	–	1	–
Yes	1.74 (0.40; 7.60)	0.4608	15.20 (1.97; 117.07)	0.0090
**Clinical presentation**		0.0029		0.0330
Mucosal	1	–	1	–
Cutaneous	5.10 (1.74; 14.93)	0.0029	10.32 (1.21; 88.20)	0.0330
**Treatment Group**		0.0379		0.0322
M + P	1	–	1	–
A + P	2.53 (1.05; 6.08)	0.0379	3.22 (1.10; 9.40)	0.0322

The influence of relevant variables was also included.

### Adverse Effects

In univariate analysis of AEs, 50% of the patients in the M+P group experienced AEs, while 90.47% of the patients in group A+P experienced AEs; the difference was significant (p = 0.003) (**Table 1**). The AEs in the M+P group were as follows, in descending order of frequency: nausea (n = 10), vomiting (n = 8), asthenia (n = 3), stomachache (n = 2), elevated transaminase levels (n =1) and dizziness (n = 1). In the A+P group, the AEs in descending order of frequency were myalgia (n = 12), elevated transaminase levels (n = 7), asthenia (n = 5), renal alterations (n = 3), elevated amylase levels (n = 2), anemia (n = 2), cardiac alterations with QTc interval enlargement (n = 1) and stomachache (n = 1). Only one patient had severe AEs in the M+P group, while 12 patients in the A+P group had at least one severe AE.

In multivariate analysis of the risk of adverse effects with the Cox regression model, patients treated with A+P had a higher risk of adverse effects (HR 3.22 (CI: 1.10 – 9.40); p = 0.032) than those treated with M+P. Additionally, patients with only CL had a higher risk of adverse effects (HR = 10.32 (CI: 1.21 - 88.20); p = 0.033) than those with ML, as did patients who had previously received treatment with antileishmanial drugs (HR = 15.20 (CI: 1.97 – 117.07); p = 0.009) (**Table 2**).

## Discussion

### Current Treatments and Supporting Evidence

Leishmaniasis, despite its increasing incidence, is included in the WHO list of neglected tropical diseases. Cochrane database systematic reviews and recent updates about interventions and treatments for both Old World and New World leishmaniasis have shown that the level of evidence in most publications was low or moderate due to methodological shortcomings that made it impossible to draw reliable conclusions (González et al., 2008; González et al., 2009; Heras-Mosteiro et al., 2017; Pinart et al., 2020). The most recent Cochrane review of New World leishmaniasis included 75 studies and concluded that intravenous meglumine antimoniate and oral miltefosine yield the best cure rates and are currently the most highly recommended treatments (Pinart et al., 2020). The association of pentavalent antimonials with drugs with immunomodulatory effects has already been tested with encouraging results (Ventin et al., 2018). It seems that penthoxyfiline reduces vacuolation of macrophages, making active drugs more effective in achieving clinical cure (de Sá Oliveira et al., 2000).

To achieve more robust evidence, it is important to standardize clinical studies about ATL, as proposed in a recent expert consensus (Olliaro et al., 2018). Harmonizing the criteria used to identify patients and to measure treatment effects can help provide more convincing evidence of treatment efficacy (Olliaro et al., 2018). In this RCT, recommendations were followed concerning the diagnostic parameters and cure criteria.

### Oral Combination Treatment

New antileishmanial drugs are rarely developed, and treatment failure due to drug resistance and host immune response is a growing concern in endemic regions (Ponte-Sucre et al., 2017). ATL, especially ML, needs to be addressed in well-designed, robust RCTs (González et al., 2008; González et al., 2009; Reveiz et al., 2013; Heras-Mosteiro et al., 2017; Carvalho et al., 2018; Pinart et al., 2020). Injectable PAs have been used since the 1940s to treat ATL and are currently the recommended first-line treatments in Brazil (Ministério da Saúde, 2017). Treatment with PAs requires daily intravenous or intramuscular injections for 20 to 30 days in areas with a high risk of ML, which means that patients have to go every day during the treatment period to a healthcare facility to receive the medication, which can impose additional burdens on the healthcare system and the patient (Burza et al., 2018; Chakravarty and Sundar, 2019; Carvalho et al., 2021).

In 2021, the estimated cost of treating patients with ML in Brazil with PAs and pentoxifylline for 30 days was US$167.66, while the cost of treatment with 150 mg/day miltefosine for 28 days was US$259.92 (Carvalho et al., 2021). However, in that cost evaluation, the eventual expenses arising from the occurrence of AEs, treatment interruptions and treatment failure with the subsequent need for other treatments were not considered, and these expenses can be relatively higher with PAs. It is known that treatment with PAs can lead to severe AEs, such as cardiac arrythmias, pancreatitis, acute renal failure, and hepatic toxicity (Chakravarty and Sundar, 2019). In addition, in that cost evaluation, the miltefosine dosage was higher than the one we propose, and the combination with pentoxifylline was not considered, which would affect the cost.

There are still few published data on the use of miltefosine for the treatment of ATL, and combined treatment with miltefosine and oral pentoxifylline in mice yielded encouraging results, with a greater reduction in viable Leishmania than achieved with miltefosine alone (Santarem et al., 2014). The idea of combining treatments to reduce AEs, increase cure rates, and reduce drug resistance is promising (Santarem et al., 2014). The possibility of using only oral drugs has the benefit of facilitating drug administration and increasing treatment adherence (Carvalho et al., 2021). As the real effect of combined treatment with miltefosine and pentoxifylline is unknown and no data have been published, we relied on a *post hoc* analysis, effect sizes and confidence intervals to assess the feasibility of future trials.

This pilot trial based on a *post hoc* analysis reflects the reported epidemiology of ATL, with a male predominance and an older age of patients with ML than of those with CL. Additionally, patients with ML have a longer delay in treatment and diagnosis than those with CL, which is characterized by visible lesions. The M+P and A+P groups were comparable, with no significant differences in demographic and epidemiological characteristics between the groups, indicating that the randomization was successful.

The treatment duration was considerably longer in the A+P group due to the higher rate of treatment interruption. These interruptions were necessary because of AEs, especially elevated levels of hepatic markers, that were significantly more frequent in the A+P group. Some of these AEs led to the need for permanent treatment suspension and treatment with LAB. This reveals the potential risks associated with this treatment (Chakravarty and Sundar, 2019).

### Curative Effect

The miltefosine dosage used in this trial was fixed at 100 mg daily, and it ranged between 1.13 mg and 1.79 mg/kg/day, which is lower than the initial dosage described as monotherapy of 2.5 mg/kg/day (Sundar et al., 2002; Sindermann et al., 2004; Soto et al., 2004; Machado et al., 2010; Chrusciak-Talhari et al., 2011) but is compatible with a more recently published dosage that yielded good results (Sampaio et al., 2019). The dosage of APs was the previously described standard and ranged from 13.96 to 19.91 mg Sb5+/kg/day.

There was no significant difference in the cure rate 90 days after the start of treatment, indicating that the proposed combination oral treatment is effective. These results were maintained 180 days after the start of treatment, indicating that the curative effect persists. There was no significant difference in the cure rate 30 days after the start of treatment, indicating that there was no difference in the speed of healing between the two evaluated treatments.

**Figure 2:**
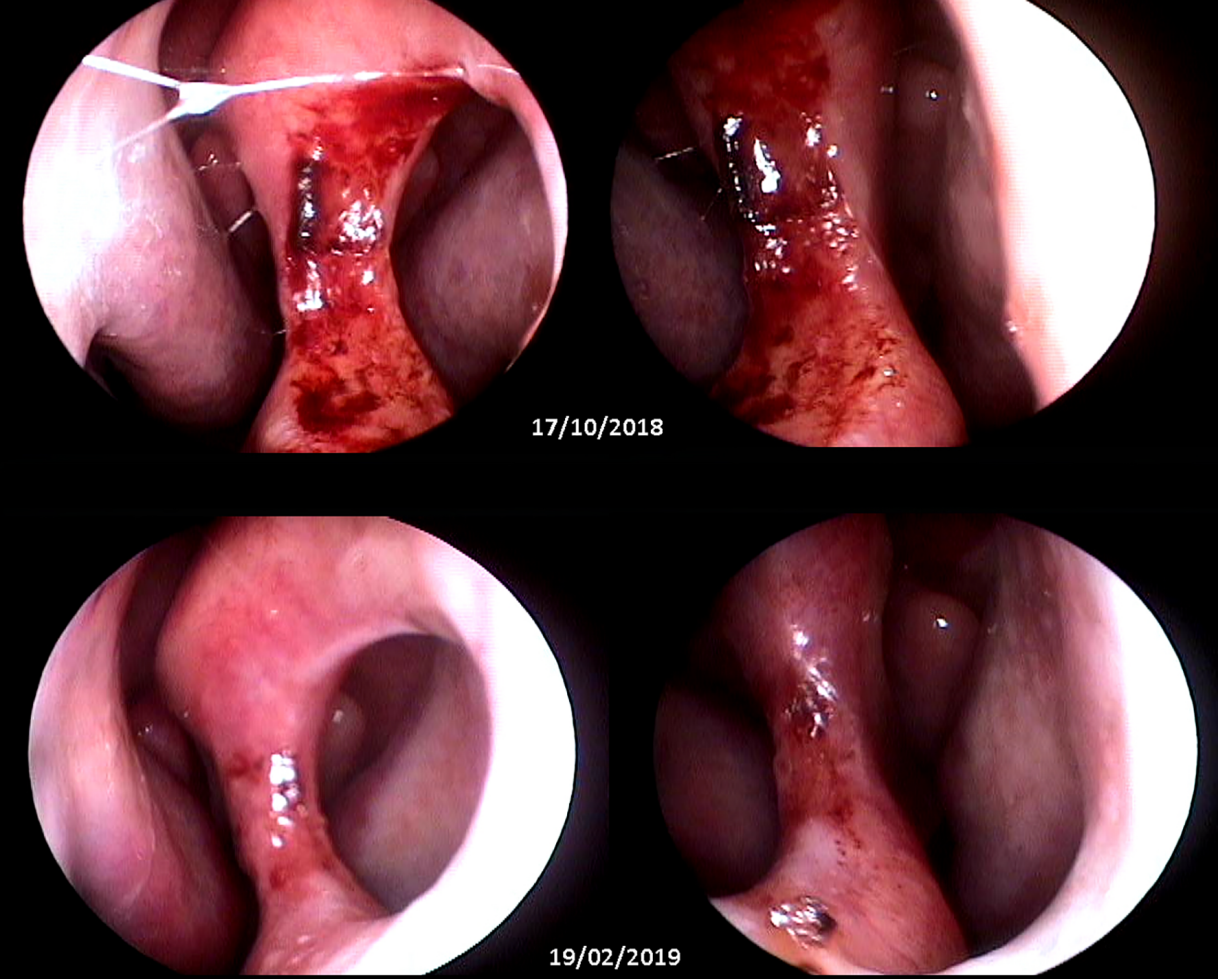
Top: wide septal perforation with infiltrated borders and granulomatous ulcerated aspect (pretreatment). Bottom: septal perforation with smooth borders without ulceration and with cicatricial aspect (90 days after treatment). Images obtained with nasofibroscopy and provided by Dr. Gustavo Subtil.

ML is more severe and difficult to treat than CL (Burza et al., 2018; Chakravarty and Sundar, 2019; Sampaio et al., 2019); therefore, it is expected that patients with CL would have a higher cure rate, even after a shorter duration of treatment (**Figure 2**). Indeed, the multivariate analysis showed that patients with cutaneous lesions had a higher cure rate in both groups. *L. (V.) braziliensis* is the main causative agent of leishmaniasis in the Americas and the pathogen most often related to ML; therefore, in areas in which this pathogen is endemic, ML should always be suspected.

Patients who had previously received antileishmanial treatment more than 6 months before entering the trial also had a higher chance of achieving a cure, possibly because previous treatment may have triggered the host immune system, leading to a better response to subsequent treatment. Another possible explanation for this result is a residual effect of previous treatment with PAs.

### Adverse Effects Analysis

The incidence of AEs was significantly higher in the A+P group (p = 0.032 in the multivariate analysis and p = 0.003 in the univariate analysis), and the most frequent AEs in this group were intense myalgia and elevated hepatic transaminase levels. The most frequent AEs in patients in the M+P group were nausea and vomiting, which usually improved after a few days, allowing the patients to complete treatment and indicating that oral treatment was well tolerated (Machado et al., 2010). In the M+P group, most of the AEs were mild (nausea and vomiting), and no patient in this group had cardiac, renal or amylase alterations, while more than half of the patients in the A+P group had at least one severe AE. Only one patient had a severe AE in the M+P group (transaminase level elevation), while 12 patients in the A+P group had severe AEs, indicating a difference in the severity of the AEs associated with the treatment regimens.

Although the mean number of days of treatment interruption was not significantly different between groups, the mean interruption duration in the M + P group was below 1 day, representing a clinically significant difference once most patients in this group did not interrupt treatment. When analyzing frequency, more patients had to interrupt treatment due to AEs in the A+P group (57% x 14%), and more patients could not finish treatment due to AEs in the A+P group (23% x 9%). Those patients were treated with intravenous LAB. Among the patients who could complete treatment, the duration of treatment was shorter in the M+P group (20-28 days *vs.* 20-94 days).

The independent characteristics that were associated with a higher risk of AEs were CL instead of ML and prior antileishmanial treatment. When we analyzed the type of AEs in the CL and ML groups, we found that in those with CL, most of the AEs were mild, such as nausea, vomiting and myalgia, while in the ML group, the AEs were more severe, such as hepatic and renal toxicity. This difference may not be related to the actual severity of the AEs and may instead reflect a reporting bias, since the patients with ML had more severe symptoms at baseline, making them less likely to report symptoms of AEs than those with CL. The increase in the occurrence of AEs associated with previous antileishmanial treatment may be due to residual toxicity from previous treatments, especially with PAs, which persist in the human body.

### Limitations

This pilot study was conducted in two referral centers for the diagnosis and treatment of LTA, which may have resulted in selection bias. Additionally, it was an open-label RCT in which both the patients and the doctors knew which medication was being used. This lack of blinding could have also led to bias. Only the statistical analysis was blinded. A convenience sample was adopted due to the limitations on the availability of miltefosine in the country. Although reduced sample sizes are frequently found in studies targeting mucous leishmaniasis because of its relative rarity, we must reinforce that treatment comparison in the multivariate analysis was followed by considerably narrow confidence intervals, enhancing confidence in the present results.

### Final Considerations

This pilot, open-label RCT with 43 ATL patients showed that the oral combination of miltefosine and pentoxifylline has a cure rate equivalent to that of traditional intravenous A+P in this population, with the additional benefit of fewer AEs. Further studies with more patients and a longer follow-up duration are needed to evaluate this promising oral treatment.

## Data Availability Statement

The raw data supporting the conclusions of this article will be made available by the authors, without undue reservation.

## Ethics Statement

The studies involving human participants were reviewed and approved by Brazilian ethics committee - Comitê de Ética em Pesquisa da Faculdade de Medicina da Universidade de Brasília CAAE: 40068714.1.1001.5558. The patients/participants provided their written informed consent to participate in this study.

## Author Contributions

SM: conducting the clinical trial, including and following up patients, gathering and analyzing data, drafting, and refining the manuscript. DB: performing mucosal clinical examination, supervising, and executing molecular examinations. BR: selecting and including patients, gathering clinical data, and performing the literature review. JM and PK: supervising patients’ diagnosis and inclusion. GF: performing all nasofibroscopy exams and analyzing and interpreting mucosal images. LP: executing the study arm in the Hospital Estadual de Doenças Tropicais Dr. Anuar Auad. CG: co-coordinating the trial design and methodology, supervising parasitological and molecular diagnoses, and critically supervising manuscript writing. RS: coordinating the study, conceiving of and designing the study, analyzing and interpreting the data, supervising the manuscript writing, reviewing the manuscript, performing the final review of the article, and acting as the beneficiary of the main financial support. All authors contributed to the article and approved the submitted version.

## Funding

Received funding from: Fundação de Apoio a Pesquisa do Distrito Federal (FAP- DF) no Edital n° 03/2016; Conselho Nacional de Desenvolvimento Científico e Tecnológico (CNPq) process 309439/2018-3 and CNPq process 307358/2017-8 researcher scolarship; and Fundação de Apoio a Pesquisa em Dermatologia (FUNADERM) de 2016.

## Conflict of Interest

The authors declare that the research was conducted in the absence of any commercial or financial relationships that could be construed as a potential conflict of interest.

## References

[B1] Bergmann J. O. de Castro Moreira Dos Santos Júnior A. Santos L. S. Silva V. M. Pompeu C. B. Arabi A. Y. M.. (2019). Accuracy of a TaqMan-based Real-Time Polymerase Chain Reaction Combined to a Novy-MacNeal-Nicolle Medium Culture for the Diagnosis of American Tegumentary Leishmaniasis. J. Eur. Acad. Dermatol. Venereol 33 (5), e188–e190. 10.1111/jdv.15440 30659669

[B2] Berman J. J. (2008). Treatment of Leishmaniasis With Miltefosine: 2008 Status. Expert Opin. Drug Metab. Toxicol. 4 (9), 1209–1216. 10.1517/17425255.4.9.1209 18721114

[B3] Burza S. Croft S. L. Boelaert M. (2018). Leishmaniasis. Lancet 392 (10151), 951–970. 10.1016/S0140-6736(18)31204-2 30126638

[B4] Carvalho J. P. Assis T. M. Simões T. C. Simões T. C. Cota G. (2021). Estimating Direct Costs of the Treatment for Mucosal Leishmaniasis in Brazil. Rev. Soc. Bras. Med. Trop. 54, e04542020. 10.1590/0037-8682-0454-2020 33533816 PMC7849328

[B5] Carvalho E. M. Llanos-Cuentas A. Romero G. A. S. (2018). Mucosal Leishmaniasis: Urgent Need for More Research. Rev. Soc. Bras. Med. Trop. 51 (1), 120–121. 10.1590/0037-8682-0463-2017 29513835

[B6] Chakravarty J. Sundar S. (2019). Current and Emerging Medications for the Treatment of Leishmaniasis. Expert Opin. Pharmacother 20 (10), 1251–1265. 10.1080/14656566.2019.1609940 31063412

[B7] Chrusciak-Talhari A. Dietze R. Talhari S. Chrusciak Talhari C. da Silva R. M. Gadelha Yamashita E. P.. (2011). Randomized Controlled Clinical Trial to Access Efficacy and Safety of Miltefosine in the Treatment of Cutaneous Leishmaniasis Caused by Leishmania (Viannia) Guyanensis in Manaus, Brazil. Am. J. Trop. Med. Hygiene 84 (2), 255–260. 10.4269/ajtmh.2011.10-0155 PMC3029178 21292895

[B8] de Sá Oliveira T. Capp Neto M. Martins B. J. Rodrigues H. A. Antonino R. M. Magalhães A. V. (2000). Action of Pentoxifylline on Experimental Cutaneous Leishmaniasis Due to Leishmania (Leishmania) Amazonensis. Mem Inst Oswaldo Cruz 95 (4), 477–482. 10.1590/S0074-02762000000400006 10904402

[B9] Dorlo T. P. C. Balasegaram M. Beijnen J. H. de Vries P. J. (2012). Miltefosine: A Review of its Pharmacology and Therapeutic Efficacy in the Treatment of Leishmaniasis. J. Antimicrobial Chemother. 67 (11), 2576–2597. 10.1093/jac/dks275 22833634

[B10] Fernández O. L. Diaz-Toro Y. Ovalle C. Valderrama L. Muvdi S. Rodríguez I.. (2014). Miltefosine and Antimonial Drug Susceptibility of Leishmania Viannia Species and Populations in Regions of High Transmission in Colombia. PloS Negl. Trop. Dis. 8 (5), e2871–e2811. 10.1371/journal.pntd.0002871 24853871 PMC4031164

[B11] Gomes C. M. Cesetti M. V. de Paula N. A. Vernal S. Gupta G. Sampaio R. N. R.. (2017). Field Validation of SYBR Green- and TaqMan-Based Real-Time PCR Using Biopsy and Swab Samples To Diagnose American Tegumentary Leishmaniasis in an Area Where Leishmania (Viannia) Braziliensis is Endemic. J. Clin. Microbiol. 55 (2), 526–534. 10.1128/JCM.01954-16 27927916 PMC5277523

[B12] Gomes C. M. de Paula N. A. de Morais O. O. Soares K. A. Roselino A. M. Sampaio R. N. R. (2014). Complementary Exams in the Diagnosis of American Tegumentary Leishmaniasis. Bras. Dermatol. 89 (5), 701–709. 2nd ed. Sociedade Brasileira de Dermatologia;. 10.1590/abd1806-4841.20142389 PMC4155947 25184908

[B13] González U. Pinart M. Rengifo-Pardo M. Macaya A. Alvar J. Tweed J. A. (2009). Interventions for American Cutaneous and Mucocutaneous Leishmaniasis. Cochrane Database Syst. Rev. 2, CD004834. 10.1002/14651858.CD004834.pub2 19370612

[B14] González U. Pinart M. Reveiz L. Alvar J. (2008). Interventions for Old World Cutaneous Leishmaniasis. Cochrane Database Syst. Rev. 4, CD004834. 10.1002/14651858.CD005067.pub3 18843677

[B15] Heras-Mosteiro J. Monge-Maillo B. Pinart M. Lopez Pereira P. Garcia-Carrasco E. Campuzano Cuadrado P.. (2017). Interventions for Old World Cutaneous Leishmaniasis. Cochrane Database Syst. Rev. 12 (12), CD005067. 10.1002/14651858.CD005067.pub5 29192424 PMC6485999

[B16] Machado P. R. Ampuero J. Guimaraes L. H. Villasboas L. Rocha A. T. Schriefer A.. (2010). Miltefosine in the Treatment of Cutaneous Leishmaniasis Caused by Leishmania Braziliensis in Brazil: A Randomized and Controlled Trial. PloS Negl. Trop. Dis. 4 (12), e912–e916. 10.1371/journal.pntd.0000912 21200420 PMC3006132

[B17] Ministério da Saúde. (2017). Brasil. Ministério Da Saúde. Secretaria de Vigilância em Saúde. Departamento de Vigilância das Doenças Transmissíveis. Manual de vigilância da leishmaniose tegumentar [recurso eletrônico] / Ministério da Saúde, Secretaria de Vigilância em Saúde, Departamento de Vigilância das Doenças Transmissíveis: il. Edição eletrônica da 2ª edição do livro: Manual de Vigilância da Leishmaniose Tegumentar Americana, atualizado. (Brasília: Ministério da Saúde), 189 p.

[B18] Olliaro P. Grogl M. Boni M. Carvalho E. M. Chebli H. Cisse M.. (2018). Harmonized Clinical Trial Methodologies for Localized Cutaneous Leishmaniasis and Potential for Extensive Network With Capacities for Clinical Evaluation. PloS Negl. Trop. Dis. Public Library Sci 12 (1), e0006141. 10.1371/journal.pntd.0006141 PMC5785032 29329311

[B19] Organização Pan-Americana da Saúde (2019). Leishmanioses: Informe Epidemiológico Nas Américas (Washington, D.C: OPAS). Available at: http://iris.paho.org/xmlui/handle/123456789/50505. Disponível em: incluir.

[B20] Pinart M. Rueda J.-R. Romero G. A. Pinzón-Flórez C. E. Osorio-Arango K. Silveira Maia-Elkhoury A. N.. (2020). Interventions for American Cutaneous and Mucocutaneous Leishmaniasis. Cochrane Database Syst. Rev. 8 (8), CD004834. 10.1002/14651858.CD004834.pub3 32853410 PMC8094931

[B21] Ponte-Sucre A. Gamarro F. Dujardin J.-C. Barrett M. P. López-Vélez R. García-Hernández R.. (2017). Drug Resistance and Treatment Failure in Leishmaniasis: A 21st Century Challenge. PLoS Negl. Trop. Dis. 11 (12), e0006052–24. 10.1371/journal.pntd.0006052 29240765 PMC5730103

[B22] Reveiz L. Maia-Elkhoury A. N. S. Nicholls R. S. Sierra Romero G. A. Yadon Z. E. (2013). Interventions for American Cutaneous and Mucocutaneous Leishmaniasis: A Systematic Review Update. PLoS One 8 (4), e61843–e61814. 10.1371/journal.pone.0061843 23637917 PMC3639260

[B23] Rugani J. N. Quaresma P. F. Gontijo C. F. Soares R. P. Monte-Neto R. L. (2018). Intraspecies Susceptibility of Leishmania (Viannia) Braziliensis to Antileishmanial Drugs: Antimony Resistance in Human Isolates From Atypical Lesions. Biomed. Pharmacother. 108, 1170–1180. 10.1016/j.biopha.2018.09.149 30372818

[B24] Sampaio R. N. R. Silva J. S. F. E. de P. C. D. R. Porto C. Motta J de O. C. D. Pereira LI de A.. (2019). A Randomized, Open-Label Clinical Trial Comparing the Long-Term Effects of Miltefosine and Meglumine Antimoniate for Mucosal Leishmaniasis. Rev. Soc. Bras. Med. Trop. SBMT 52 (5), 701–708. 10.1590/0037-8682-0292-2018 30942258

[B25] Santarem A. A. A. Greggianin G. F. Debastiani R. G. Ribeiro J. B. P. Polli D. A. Sampaio R. N. R. (2014). Effectiveness of Miltefosine-Pentoxifylline Compared to Miltefosine in the Treatment of Cutaneous Leishmaniasis in C57Bl/6 Mice. Rev. Soc. Bras. Med. Trop. SBMT 47 (4), 517–520. 10.1590/0037-8682-0202-2013 25229296

[B26] Sindermann H. Croft S. L. Engel K. R. Bommer W. Eibl H. J. Unger C.. (2004). Miltefosine (Impavido): The First Oral Treatment Against Leishmaniasis. Med. Microbiol. Immunol. 193 (4), 173–180. 10.1007/s00430-003-0201-2 14513375

[B27] Soto J. Arana B. A. Toledo J. Rizzo N. Vega J. C. Diaz A.. (2004). Miltefosine for New World Cutaneous Leishmaniasis. Clin. Infect. Dis. 38 (9), 1266–1272. 10.1086/383321 15127339

[B28] Sundar S. Jha T. K. Thakur C. P. Engel J. Sindermann H. Fischer C.. (2002). Oral Miltefosine for Indian Visceral Leishmaniasis. N. Engl. J. Med. 347 (22), 1739–1746. 10.1056/NEJMoa021556 12456849

[B29] Ventin F. Cincurá C. Machado P. R. L. (2018). Safety and Efficacy of Miltefosine Monotherapy and Pentoxifylline Associated With Pentavalent Antimony in Treating Mucosal Leishmaniasis. Expert Rev. Anti-infective Ther. 0 (0), 1–26. 10.1080/14787210.2018.1436967 29411659

